# Gut microbiota from patients with Parkinson’s disease causes motor deficits in honeybees

**DOI:** 10.3389/fmicb.2024.1418857

**Published:** 2024-07-12

**Authors:** Jiaqi Zeng, Yiyuan Li, Jingshuang Yan, Ruqi Chang, Mengqi Xu, Guanzhou Zhou, Jie Meng, Di Liu, Zhiqi Mao, Yunsheng Yang

**Affiliations:** ^1^Microbiota Division, Department of Gastroenterology and Hepatology, First Medical Center, Chinese PLA General Hospital, Beijing, China; ^2^Medical School of Chinese PLA, Beijing, China; ^3^Department of Neurosurgery, First Medical Center, Chinese PLA General Hospital, Beijing, China; ^4^National Clinical Research Center for Geriatric Diseases, Chinese PLA General Hospital, Beijing, China

**Keywords:** Parkinson’s disease, gut microbiota, honeybee model, rotenone, microbiota-gut-brain axis

## Abstract

**Objective:**

Parkinson’s disease (PD) is possibly caused by genetic factors, environmental factors, and gut microbiota dysbiosis. This study aims to explore whether the microbiota contributes to the behavior abnormalities of PD.

**Methods:**

We transplanted gut microbiota from patients with PD or healthy controls (HC) into microbiota-free honeybees. We also established two more groups, namely the rotenone (ROT) group, in which PD-like symptoms of honeybees were induced by rotenone, and the conventional (CV) group, in which honeybees were colonized with conventional gut microbiota. The climbing assay was performed to assess the motor capabilities of honeybees. Histopathological examination was conducted to evaluate the integrity of gut mucosa. Tyrosine hydroxylase (TH) gene expression levels and dopamine (DA) concentrations in the brain were also examined. Additionally, metagenomics and full-length 16S rRNA analyses were performed to identify alterations in gut microbiota profiles, both in PD patients and honeybees.

**Results:**

Honeybees in the PD and ROT groups exhibited slower climbing speeds, downregulated TH gene expression, and impaired gut barriers. Both the HC and PD groups of honeybees successfully harbored a portion of gut microbiota from corresponding human donors, and differences in microbial composition were identified. *Morganella morganii* and *Erysipelatoclostridium ramosum* exhibited significantly increased relative abundance in the HC group, while *Dorea longicatena*, *Collinsella aerofaciens*, *Lactococcus garvieae*, *Holdemanella biformis*, *Gemmiger formicilis*, and *Blautia obeum* showed significantly increased relative abundance in the PD group. Functional predictions of microbial communities in the PD group indicated an increased synthesis of hydrogen sulfide and methane.

**Conclusion:**

A novel PD model was induced in honeybees with rotenone and gut microbiota from PD patients. This study linked PD-related behaviors to altered gut microbiota, highlighting a potential gut microbiota-brain axis involvement in PD pathogenesis. We identify previously unrecognized associations of *Dorea longicatena*, *Collinsella aerofaciens*, *Lactococcus garvieae*, *Holdemanella biformis*, *Gemmiger formicilis*, and *Blautia obeum* with PD. Additionally, pathways related to hydrogen sulfide and methane synthesis have been previously suggested as potential contributors to the development of PD, and our research further supports this hypothesis.

## Introduction

Parkinson’s disease (PD), the second most common neurodegenerative disorder, is a progressive and degenerative disease characterized by motor and non-motor symptoms (Hou et al., 2021). The classic motor symptoms include resting tremors, bradykinesia, postural instability, and rigidity, whereas non-motor symptoms involve a wide range of features such as depression, anxiety, sleep disturbance, and gastrointestinal complaints including constipation, abdominal bloating, and dysphagia (Hayes, 2019). The onset of motor symptoms is primarily associated with the loss of dopaminergic neurons in the substantia nigra (Scheperjans et al., 2015). In dopaminergic neurons, tyrosine hydroxylase (TH) is the rate-limiting enzyme in dopamine synthesis (Kostrzewa et al., 2005) and is used as a marker for the activity of the dopaminergic neurons (Hou et al., 2021).

Previously, PD was thought to be caused primarily by a combination of genetic and environmental factors (Kalia and Lang, 2015), but recent studies have revealed that gut microbiota might play an important role in the pathogenesis of PD. The composition of gut microbiota was altered in PD patients in comparison to healthy controls (HC) (Scheperjans et al., 2015; Yan et al., 2021), and gut microbiota restoration via fecal microbiota transplantation (FMT) as well as probiotic supplementation could improve the motor and non-motor symptoms of PD patients (Kuai et al., 2021; Mirzaei et al., 2022). In a well-established mouse model of PD by Sampson et al. (2016), antibiotic treatment alleviated the pathophysiology of PD, while microbial re-colonization had a promoting effect on the disease progression. The interplay among the gut, gut microbiota, and the brain has been illustrated and termed the microbiota-gut-brain axis (Cryan and Dinan, 2012). Given the above-mentioned evidence, gut microbiota may influence bidirectional gut-brain signaling pathways through the microbiota-gut-brain axis (Zeng et al., 2022).

Honeybees, with their high accessibility, short lifespan, easy maintenance, and ease of obtaining a large sample of microbiota-free subjects, serve as a potentially ideal model for studying the relationship between gut microbiota and diseases (Wang et al., 2018; Zheng et al., 2018). In our previous research, we successfully constructed honeybee models of autism (Li et al., 2023) and enteritis (Chang et al., 2022). Furthermore, the reduction in dopamine concentration is a characteristic feature of PD, and dopamine also modulates honeybee behaviors and motor abilities (Maze et al., 2006; Raza and Su, 2020). Moreover, genes associated with honeybee motor behaviors share homology with human PD-related genes (Alaux et al., 2009). Honeybees exhibit phototaxis by climbing toward light sources (Yamaguchi and Heisenberg, 2011). Damage to the honeybee’s brain may result in impaired balance and slower movement, consequently leading to an extension of climbing time. Thus, the climbing assay can be employed to assess their locomotion (Luo et al., 2021). Hence, we hypothesized the feasibility of constructing a PD honeybee model. Additionally, rotenone, a pesticide that inhibits mitochondrial complex I, can induce symptoms resembling PD in mice and *Drosophila melanogaster* (Coulom and Birman, 2004; Chia et al., 2020), which are characterized by significant locomotor impairments and degeneration of dopaminergic neurons. According to our preliminary experiments, we have identified a sublethal dose of rotenone suitable for constructing a PD honeybee model.

Studies have reported that FMT from patients with schizophrenia (Zhu et al., 2020) or autism spectrum disorder (Sharon et al., 2019) in germ-free mice could induce specific behavioral symptoms of the corresponding disease. Therefore, to investigate whether dysbiosis of the gut microbiota can directly induce PD symptoms and pathology, and to investigate the underlying role of pathogenic microorganisms, we transplanted the gut microbiota from PD patients into microbiota-free honeybees. We observed and compared the motor abilities of four groups: honeybees with conventional (CV) gut microbiota, honeybees transplanted with gut microbiota from healthy individuals or PD patients, and honeybees treated with rotenone.

## Methods

### Fecal microbiota preparation

Fecal samples were collected from PD patients diagnosed at the Chinese PLA General Hospital and from healthy donors matched as closely as possible to PD patients. The exclusion criteria for PD subjects were as follows: (1) atypical or secondary parkinsonism; (2) use of probiotics or antibiotics within 3 months before sample collection; (3) history of chronic gastrointestinal diseases; (4) unstable medical, neurological, or psychiatric conditions. The inclusion criteria for healthy subjects were as follows: (1) normal blood tests; (2) no neurological diseases; (3) no gastrointestinal diseases, including irritable bowel syndrome, inflammatory bowel disease, colitis, cancer, etc.; (4) no use of probiotics or antibiotics within 3 months before sample collection. This study was approved by the Ethics Committee of Chinese PLA General Hospital (S2019-061-02).

The fresh stool samples were suspended with sterile phosphate-buffered saline (PBS, 7.5 mL/g of feces) and centrifuged at 800 g for 5 min at 4°C. The supernatants were mixed with an equal volume of 40% glycerol-PBS, resulting in a final glycerol concentration of 20%, and vortexed for 5 min. The procedures were conducted in a biosafety cabinet. The fecal suspensions containing microbiota were stored at −80°C before being used for transplantation.

### Honeybee rearing and experimental grouping

Honeybees (worker bees) were purchased from Shunyi County of Beijing in July 2022. Microbiota-free bees were obtained as described by Zhang et al. (2022a). Briefly, late-stage bee pupae were manually removed from mature brood frames and placed in a sterilized feeding box. The bees then naturally emerged at a temperature of 35°C and 50% humidity. After emergence, bees (0 days old) were fed in separate sterilized cup cages with sterile sucrose syrup (50%, wt/vol). After 24 h, guts from random 1-day-old bees are collected for sterility verification using blood agar plates.

One-day-old bees were then divided into four groups: (1) CV bees; (2) PD bees; (3) HC bees; and (4) rotenone (ROT) bees. A total of 75 bees were allocated to each group, and every 25 bees were kept in one cup cage. For the CV group, each cup cage of bees was fed with a mixture consisting of 10 μL of gut homogenates, which included normal gut microbiota from freshly collected guts of nurse bees from their hives of origin, 900 μL of 1 × PBS, 600 μL of sterile sucrose solution (50%, wt/vol), and 500 μL of sterile pollen. For the PD group, each cup cage of bees was fed with a 2 mL mixture, consisting of 1 mL of fecal suspensions containing microbiota from PD patients and 1 mL of sterile sucrose solution. Likewise, for the HC group, each cup cage of bees was fed with a 2 mL mixture, consisting of 1 mL of fecal suspensions containing microbiota from healthy subjects and 1 mL of sterile sucrose solution. For the ROT group, each cup cage of bees was fed with 2 mL of rotenone suspension (500 μM, dispersed in sucrose solution). After 24 h, CV, PD, and HC bees were then fed with sterile sucrose solution and pollen until day 8, while after 6 days, RT bees were fed with sterile sucrose solution and pollen until day 8. Animal maintenance and experimental procedures were carried out in compliance with the Guide for the Care and Use of Laboratory Animals.

### Climbing assays

The climbing assays were performed on day 8. The behavioral apparatus was a black acrylic sand-textured box (55 cm in length, 35 cm in width, 4 cm in height) containing 10 lanes (50 cm long), with LED lights placed at the endpoint, as described by Zaluski et al. (2015) with some modifications. The tests were conducted in the dark. The apparatus was inclined at a 45° angle, with each bee placed at the starting point of each lane. After the partition was removed, the bees climbed upward due to phototaxis. The time taken to reach the endpoint was recorded. All tests were performed in triplicate.

### Tissue collection

The entire gut and brain of bees were collected after behavioral tests. The dissection method for bees followed the procedure described by Zhang et al. (2022a). Briefly, microscopic dissection was performed to collect the entire gut and brain with non-brain structures such as compound eyes, pigments, and glands removed. The dissected tissues were snap-frozen in liquid nitrogen and stored at −80°C.

### RNA extraction and RT-qPCR

Brain tissues were homogenized using an electric tissue homogenizer (TIANGEN). Total RNA was extracted utilizing the FastPure^®^ Cell/Tissue Total RNA Isolation Kit V2 (Vazyme, China) under the manufacturer’s instructions. The RNA concentration and purity were then determined using a NanoDrop spectrophotometer. Complementary DNA (cDNA) was synthesized from the extracted RNA using the HiScript^®^ III RT SuperMix for qPCR (+gDNA wiper) (Vazyme, China). RT-qPCR was performed to assess tyrosine hydroxylase (TH) gene expression levels using the ChamQ Universal SYBR qPCR Master Mix (Vazyme, China) on a Real-Time PCR Instrument (Thermo Fisher Scientific, Waltham, MA, United States). RT-qPCR was performed under the following conditions: (1) initial denaturation: 95°C for 30 s. (2) Cycling (40 cycles): denaturation: 95°C for 10 s; annealing: 60°C for 30 s. (3) Melting curve analysis: 95°C for 15 s, 60°C for 60 s, 95°C for 15 s. The following primers were used: TH: Forward: 5′-AACCGCGAGATGTTCGCTAT-3′, Reverse: 5′-AGTCCAGCGTCATTCGCC-3′; Actin: Forward: 5′-TGCCAACACTGTCCTTTCTG-3′, Reverse: 5′-AGAATTGACCCACCAATCCA-3′. The relative gene expression levels were normalized to the *Apis mellifera* actin gene and calculated using the 2^−ΔΔCT^ method (Song et al., 2022). Each RT-qPCR experiment was conducted in triplicate to ensure reproducibility.

### Dopamine ELISAs

The dopamine concentrations in the brain homogenates were determined using the Insect DA ELISA Kit (Mlbio, China) following the manufacturer’s instructions. Standard curves were generated using known concentrations of dopamine provided in the kit.

### Hematoxylin-eosin staining

The gut tissues of honeybees were fixed in 4% paraformaldehyde for 24 h. Following fixation, tissues underwent dehydration through a graded series of ethanol solutions. Subsequently, the tissues were cleared using xylene. After dehydration and transparency, the tissues were embedded in paraffin. Paraffin-embedded tissues were sectioned at 4 μm using a microtome. The sections were mounted on glass slides. Deparaffinization in xylene and rehydration through a graded series of ethanol solutions were performed on the sections. Hematoxylin was used for staining nuclei, followed by differentiation using acid alcohol. Eosin was applied for cytoplasmic staining. Dehydration was performed using graded ethanol solutions, and the sections were cleared with xylene. Finally, the sections were coverslipped using a neutral resin. Microscopic observation was conducted using an upright microscope (Leica, Germany) to examine the morphological structure of the gut tissues.

### Metagenomic sequencing of human fecal samples and taxonomic identification

Fecal samples from human subjects were analyzed by metagenomics sequencing on the Illumina NovaSeq platform. Microbiota analyses were executed on the Majorbio Cloud Platform.^1^ The raw sequencing data underwent quality control using Fastp (v0.20.0). Alignment to the host (*Homo sapiens*) genome was performed using BWA (v0.7.17), enabling the identification and removal of host-derived sequences. The host genome was referenced using the Ensembl database (Release 104) for precise mapping and subsequent analyses. *De novo* assembly of the sequencing data was conducted using MEGAHIT (v1.1.2). Prodigal (v2.6.3) was employed to predict open reading frames (ORFs) within the assembled contigs obtained from *de novo* assembly. The gene sequences predicted from the samples underwent clustering using CD-HIT software (v4.7). Employing BLASTP (Version 2.2.28+), the non-redundant gene set was aligned against the NR (non-redundant) protein database to obtain species annotation results. Alpha and beta diversity indexes were calculated using QIIME2 software. Linear Discriminant Analysis Effect Size (LEfSe) was applied to identify species features contributing significantly to group differences.

### Full-length 16S rRNA sequencing and analysis

Microbial DNA was extracted from the gut contents of honeybees. Full-length 16S rRNA gene sequences were amplified using primers designed to span the entire gene. Subsequently, the amplified 16S rRNA gene products underwent library preparation using PacBio’s SMRTbell prep kit 3.0. Sequencing was performed on the PacBio platform. Raw data were processed using PacBio’s SMRTLink v11.0 software, specifically utilizing the Circular Consensus Sequencing (CCS) module, resulting in high-fidelity reads in fastq format. Denoising was performed using the DADA2 method to optimize and obtain Amplicon Sequence Variants (ASVs).

Microbiota analyses were executed on the Majorbio Cloud Platform (see text footnote 1). ASVs were taxonomically annotated using the QIIME2 bioinformatics platform (version 2022.2). The Ribosomal Database Project (RDP) Classifier served as the annotation method, with the nt_v20221012/16s_bacteria database used as the reference. Alpha and beta diversity indexes were calculated using QIIME2 software. LEfSe was applied to identify species features contributing significantly to group differences, utilizing a linear discriminant analysis (LDA) threshold of 2.5. Functional genes were annotated to the Kyoto Encyclopedia of Genes and Genomes (KEGG) database using PICRUSt2 (version 2.2.0-b). The relative abundance of the KEGG orthologs (KO) was further analyzed by the Wilcoxon rank-sum test.

### Statistical analysis

Data obtained from the honeybee climbing assays, as well as the brain dopamine and TH gene relative expression measurements across different groups, were processed and analyzed using R software (Version 4.2.0). The normality of data was assessed using the Shapiro–Wilk test, and the homogeneity of variance was evaluated with Bartlett’s test. Data exhibiting normal distribution and homogeneity of variance were expressed as mean ± standard deviation and analyzed using the independent samples *t*-test (for comparisons between two groups) or analysis of variance (for comparisons between multiple groups). In cases of non-normally distributed or heteroscedastic data, median and interquartile ranges were utilized, and comparisons were made using the Mann–Whitney *U* test (for comparisons between two groups) or Kruskal–Wallis test (for comparisons between multiple groups) and the Dunn’s test is conducted as a post-hoc test. Statistical significance was defined as a *p*-value less than 0.05.

## Results

### PD microbiota induces motor dysfunction in bees

**Figure 1:**
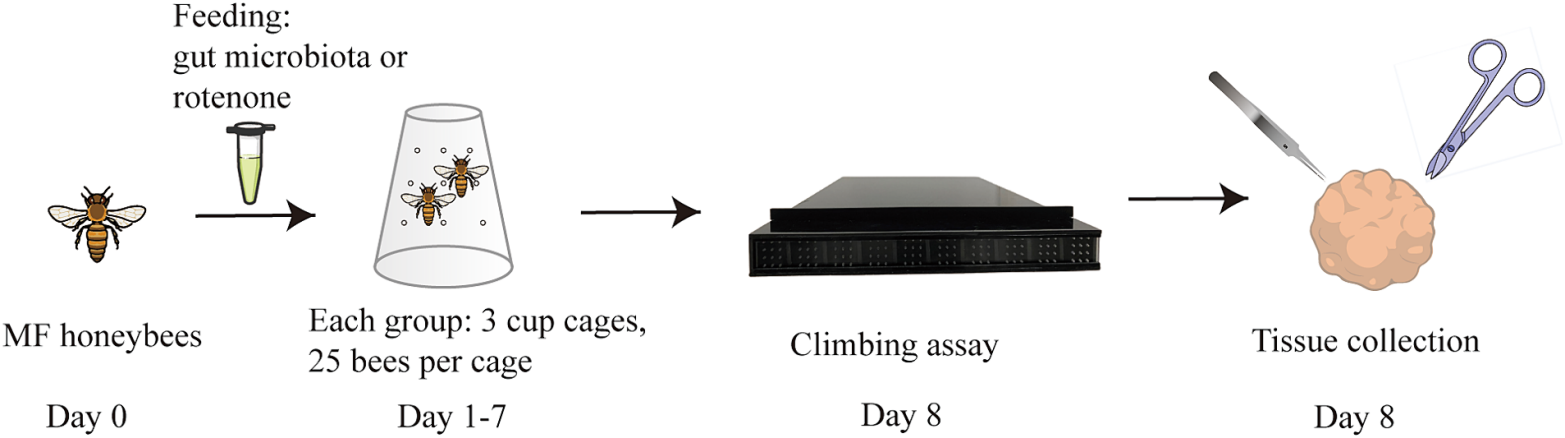
Flowchart of the study. One-day-old microbiota-free (MF) honeybees were divided into four groups: (1) PD group: colonized with gut microbiota from PD patients (5 patients, 5 PD subgroups); (2) HC group: colonized with gut microbiota from healthy controls (3 individuals, 3 HC subgroups); (3) CV group: colonized with the gut microbiota of normal honeybees; (4) ROT group: fed with rotenone. Behavioral tests were conducted on day 8, followed by tissue collection.

**Figure 2:**
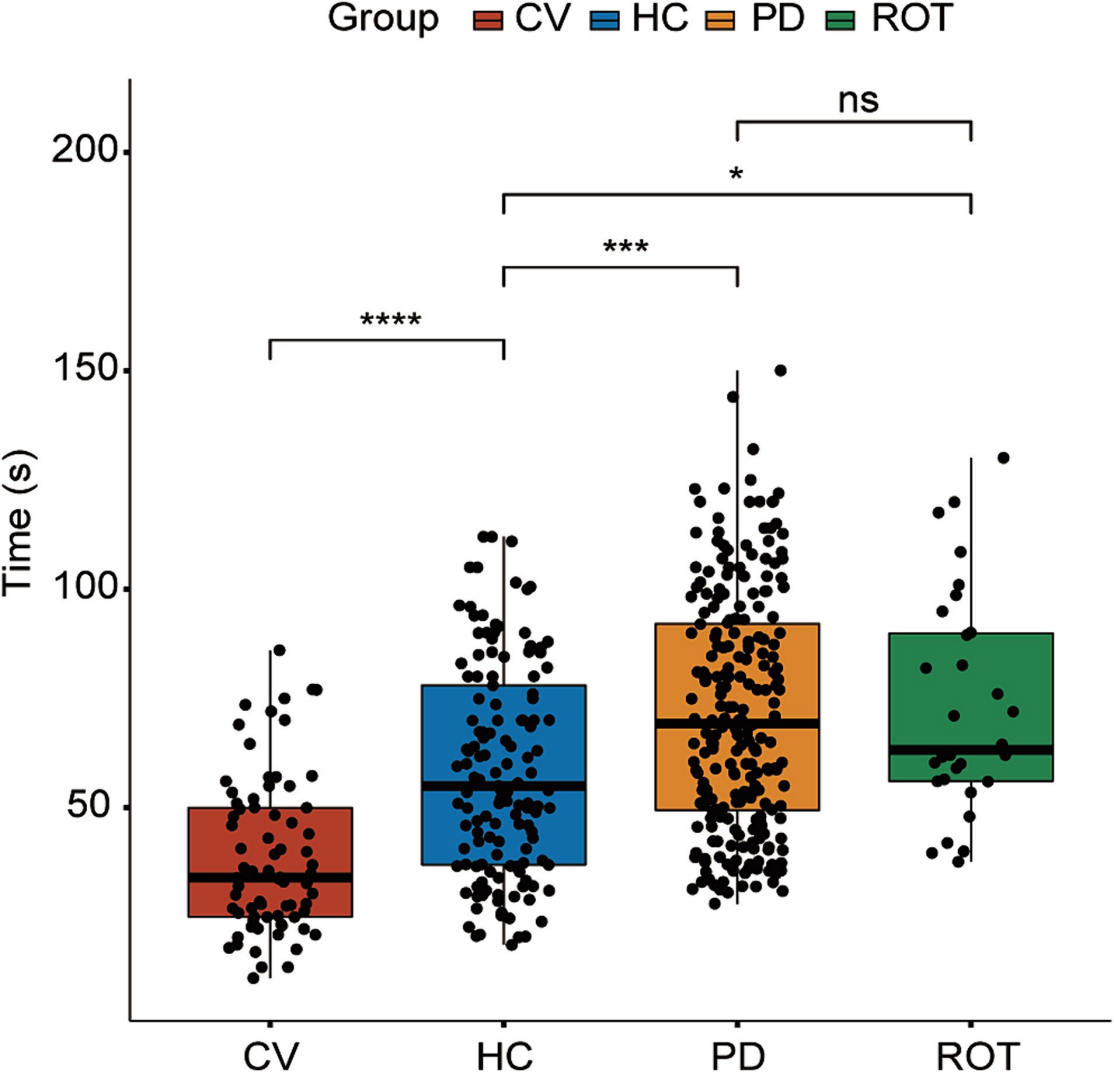
Results of the behavioral test. HC: *n* = 137, CV: *n* = 74, PD: *n* = 239, ROT: *n* = 30. ns, not significant, ^*^p < 0.05, ^***^p < 0.001, and ^****^p < 0.0001. Time (s): time taken by the bees to climb a 50 cm-long lane. Statistical analysis was performed using the Kruskal–Wallis test followed by Dunn’s post-hoc test.

The flowchart of the study is illustrated in Figure 1. To investigate the causal relationship between gut microbiota and the onset of PD, we compared the behavioral performance of PD-transplanted bees with those transplanted with HC microbiota, CV bees, and ROT bees. The results revealed that the CV group exhibited the shortest median running time (time taken by the bees to climb a 50-cm-long lane), followed by the HC group, and the PD and ROT groups showed the longest running time, as shown in Table 1. Within the PD group, the running time of bees exhibited a positive correlation with the corresponding PD patient’s age and disease severity overall, but the differences were not statistically significant (*p* > 0.05). Upon combining the five PD groups and three HC groups, respectively, for statistical analysis, we observed that the running speed of the CV group was significantly faster than that of the HC group (*p* < 0.0001). Additionally, the running speed of the HC group was significantly faster than that of the PD (*p* = 0.0001) and ROT groups (*p* = 0.0493), with no statistically significant difference found between the running speeds of the PD and ROT groups (see Figure 2).

**Table 1:** Results of the climbing assays of honeybees.

Group	Time (second), median	Age	Disease duration	Hoehn–Yahr stage	UPDRS part III
CV	34	NA	NA	NA	NA
HC1	49	63	NA	NA	NA
HC3	56.5	67	NA	NA	NA
HC2	58	50	NA	NA	NA
PD3	60	53	8	1.0	29
ROT	63.25	NA	NA	NA	NA
PD1	65.33	76	6	4.0	45
PD5	68.5	63	4	3.0	37
PD2	69.5	71	6	4.0	80
PD4	82.5	71	6	4.0	53

### PD microbiota reduces brain TH expression in bees

**Figure 3:**
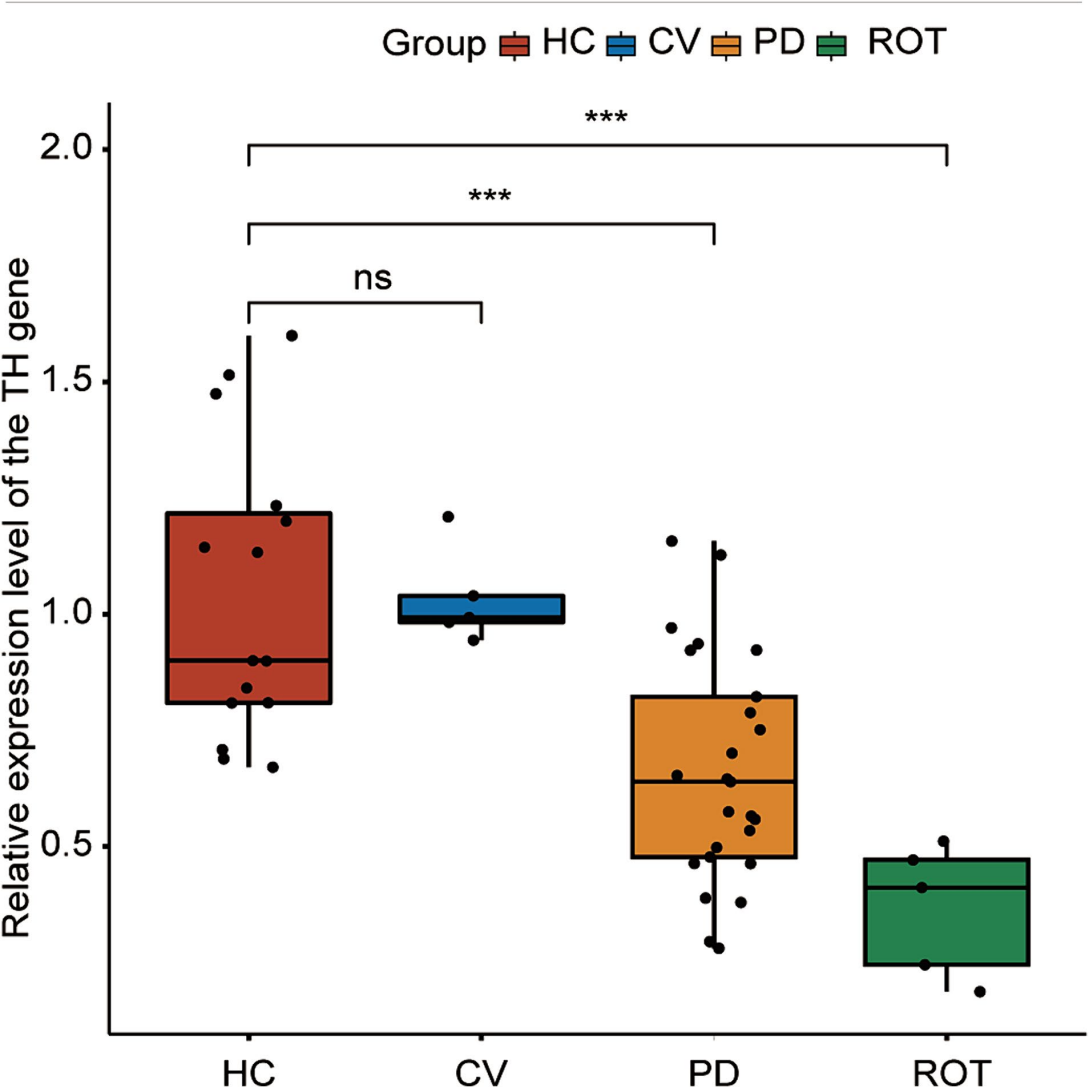
Expression of the tyrosine hydroxylase (TH) gene in the brain of honeybees, as measured by RT-qPCR. HC: *n* = 15, CV: *n* = 5, PD: *n* = 25, ROT: *n* = 5. ns, not significant, ^***^p < 0.001. Statistical analysis was conducted using the Mann–Whitney *U* test.

RT-qPCR was performed to assess the expression levels of the TH gene in the brains of honeybees. We combined the five PD groups and three HC groups, respectively, for statistical analysis. The expression levels of the TH gene were significantly downregulated in both the PD (*p* = 0.00084) and ROT (*p* = 0.00013) groups compared to the HC group. Interestingly, no significant difference was observed between the HC group and the CV group. The results are presented in Figure 3.

We further performed dopamine ELISAs to investigate the dopamine concentrations in the brain among groups. However, although the dopamine concentrations in the HC and CV groups appeared higher than those in the PD and ROT groups, the differences were not statistically significant (Supplementary Figure S1).

### PD microbiota disrupt gut barriers in bees

**Figure 4:**
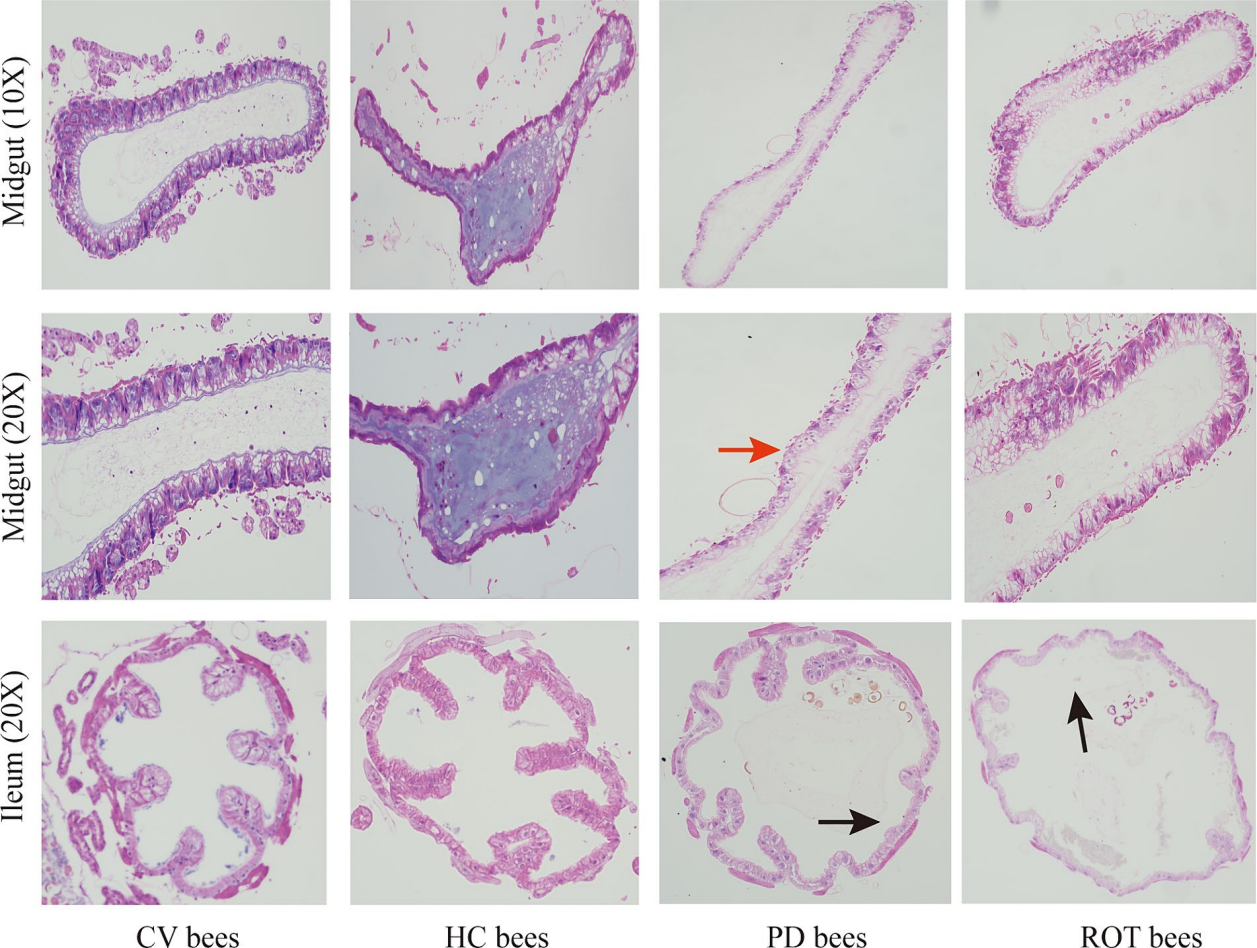
Hematoxylin and eosin staining on the midgut and ileum of honeybees. The red arrow indicates epithelial cell swelling, and the black arrows indicate dilation of the intestinal lumen and disruption of villous structures.

We performed hematoxylin and eosin (HE) staining on the midgut and ileum of honeybees. The histopathological examination of gut tissues revealed alterations indicative of gut barrier disruption in PD bees and ROT bees, compared to CV bees and HC bees (Figure 4). The observed changes included epithelial cell swelling, dilation of the intestinal lumen, and disruption of villous structures. These pathological findings collectively suggest compromised gut barrier function, highlighting the potential implications for gut health and homeostasis.

### Gut microbiota dysbiosis in bees

We collected fecal samples from eight human donors, specifically five patients with PD and three matched healthy controls. Detailed demographic and clinical information were collated in Supplementary Table S1. The beta diversity analysis of the gut microbiota in human donors revealed that the microbial composition of patients with PD was different from that of HC subjects at the genus and species levels (Supplementary Figure S2).

**Figure 5:**
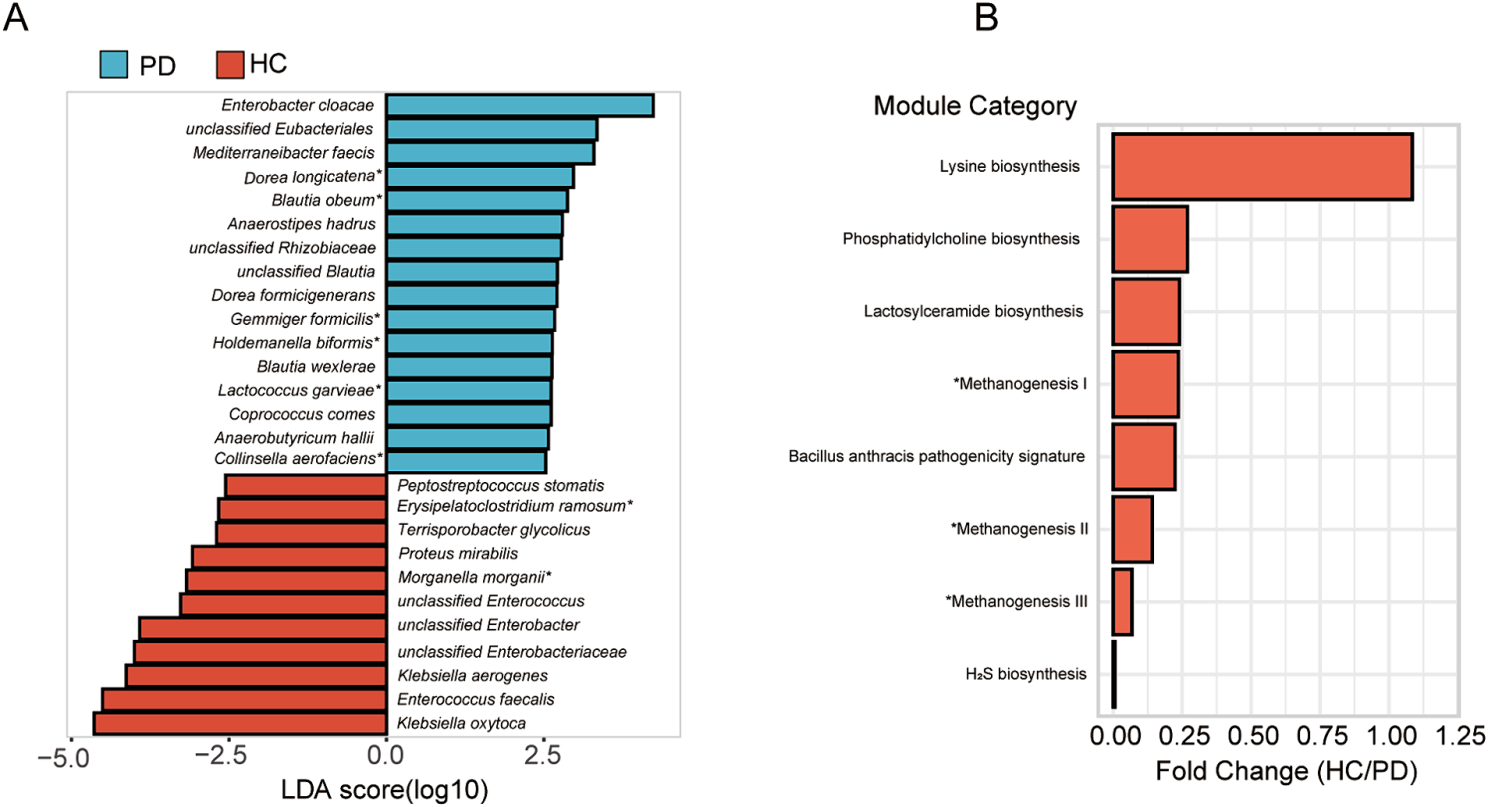
Differential taxonomic and functional analysis of gut microbiota in honeybees. **(A)** LEfSe analysis of microbiota composition between PD and HC bees. ^*^Eight species also exhibited differences in the microbial population of human donors. **(B)** Functional prediction analysis unveiling altered metabolic pathways. ^*^Methanogenesis I: CO_2_ ⇒ methane; methanogenesis II: methylamine/dimethylamine/trimethylamine ⇒ methane; methanogenesis III: methanol ⇒ methane.

In the gut microbiota analysis of honeybees, alpha diversity analysis showed that microbial richness indices (Chao index) were significantly higher in CV bees compared to HC bees, and diversity indices (Shannon index) were significantly higher in CV bees compared to PD bees (Supplementary Figure S3). The beta diversity analysis showed that the microbial composition among CV, HC, and PD bees was significantly different at the genus and species levels (Supplementary Figure S4). We used a logarithmic LDA score threshold of 2.5 to identify significant taxonomic differences between the PD and HC bees. The results of LEfSe analysis revealed a significant difference in fecal microbiota composition between the PD and HC bees, as illustrated in Figure 5A. In these differentially abundant microorganisms, eight species also exhibited differences in the microbial population of human donors, including *Morganella morganii* and *Erysipelatoclostridium ramosum*, which showed significantly increased relative abundance in the HC group, as well as *Dorea longicatena*, *Collinsella aerofaciens*, *Lactococcus garvieae*, *Holdemanella biformis*, *Gemmiger formicilis*, and *Blautia obeum*, which exhibited significantly increased relative abundance in the PD group.

By the functional prediction analysis of the 16S rRNA gene sequence data, we found that in the HC group, there was an observed increase in the predicted functional pathway related to lysine biosynthesis, suggesting an upregulation of processes associated with lysine production. Conversely, in the PD group, an enhancement in the predicted pathways related to methane synthesis, hydrogen sulfide (H_2_S) synthesis, and pathogenicity was identified (Figure 5B).

## Discussion

The microbiota-gut-brain axis plays a crucial role in the pathophysiology of PD, and our research further provides evidence supporting this perspective. We obtained normally developed CV bees whose guts were colonized with conventional microbiota, humanized honeybees whose guts were re-colonized with the fecal microbiota of PD patients or healthy controls, and ROT bees treated with rotenone. We observed that the conventional microbiota of wild honeybees and a fraction of the donor’s microbiota successfully colonized the honeybee’s digestive tract, manifesting distinct microbial enrichment patterns among the three groups of honeybees. Phototaxis is a behavior observed in many insects (Yamaguchi and Heisenberg, 2011), such as honeybees and *Drosophila*. Honeybees climb towards a light source due to phototaxis, and damage to the honeybee’s brain would result in impaired balance and slowness of movement, consequently leading to an extension of climbing time. Thus, the climbing assay can be employed to assess their locomotion (Luo et al., 2021). In this study, we hypothesized that a significant decrease in the motor abilities of honeybees could in part parallel motor symptoms of PD. As expected, CV bees displayed the highest motor capabilities, followed by HC bees with a reduced motor capacity. Nevertheless, HC bees exhibited superior motor abilities compared to PD and ROT bees. *Drosophila* has been used as a PD model for over two decades (Feany and Bender, 2000; Rahul and Siddique, 2022). Exposure to rotenone can induce motor impairments and a dramatic and selective loss of dopaminergic neurons in all of the brain clusters of *Drosophila* (Coulom and Birman, 2004). The results of our study indicated that a sublethal dose of rotenone could also impair the motor ability of honeybees, suggesting that rotenone may also cause cell apoptosis, oxidative damage, and trigger endoplasmic reticulum stress in honeybees (Coulom and Birman, 2004). The results of the climbing assay also indicated the feasibility of utilizing the compromised climbing ability in honeybees as a reflection of motor symptoms of PD.

At the molecular level, TH gene expression levels were significantly downregulated in both the PD and ROT groups compared to the HC group. These expression patterns aligned with the outcomes of the behavior phenotype. In dopaminergic neurons, TH is the rate-limiting enzyme in dopamine synthesis (Kostrzewa et al., 2005) and is used as a marker of the dopaminergic neurons (Hou et al., 2021). In invertebrates, extensive research indicates that dopamine signaling significantly influences locomotion (Chase et al., 2004; Lima and Miesenböck, 2005; Draper et al., 2007). Moreover, dopamine impacts locomotor behavior at various levels, encompassing modulation of the neuromuscular junction, regulation of central pattern generators, and modulation of general arousal levels (Dasari and Cooper, 2004; Andretic et al., 2005; Puhl and Mesce, 2008). A study by Mustard et al. (2010) revealed that dopamine modulates various motor behaviors in honeybees, encompassing walking, halting, upside-down movement, grooming, flying, and fanning. In our study, TH gene expression levels were reduced in both the PD and ROT groups, indicating the potential use of TH gene expression as an indicator of impaired motor function. Nevertheless, the dopamine levels measured by ELISAs did not exhibit a significant difference among the four groups. Several factors may contribute to such results. Firstly, a previous study found that levels of dopamine are lower in microbiota-free honeybees (Zhang et al., 2022b). However, our study found that the microbiota-free ROT group did not show a significant reduction in the levels of dopamine, suggesting potentially insufficient sample size. Secondly, the concentration of dopamine is influenced by its metabolism and reuptake. Even with a reduction in TH gene expression, mechanisms such as increased dopamine metabolism or slowed reuptake by dopamine transporter (DAT) may exist, leading to the relatively stable maintenance of dopamine levels (Klein et al., 2019). Thirdly, there might be a time lag between changes in TH gene expression and dopamine levels. TH, as the rate-limiting enzyme in dopamine synthesis, may not immediately reflect alterations in dopamine levels. It is possible that after a decrease in TH gene expression, a certain amount of time is needed for noticeable changes in dopamine levels to occur. Fourthly, PD involves neurotransmitters beyond dopamine. Abnormalities in non-dopaminergic neurotransmitters, such as acetylcholine, glutamate, norepinephrine, and serotonin, also contribute to the manifestation of PD symptoms (Kalia and Lang, 2015). Further studies should be undertaken to investigate the aforementioned possibilities.

The histology of guts revealed that gut barriers were disrupted in PD bees and ROT bees, causing enteritis-like pathology. A similar pathology was identified in our previous honeybee model of enteritis (Chang et al., 2022). In patients with PD, elevated levels of pro-inflammatory cytokines including TNF-α, IF-γ, IL-6, and IL-1β have been detected in both the colons and peripheral blood (Devos et al., 2013; Qin et al., 2016). Gastrointestinal inflammation is implicated in the increased intestinal permeability. Traversed gut microbiota and their metabolites may induce the production of proinflammatory cytokines in the enteric nervous system and aggregation of pathological α-synuclein (Zeng et al., 2022). Instead of synthesizing pro-inflammatory cytokines in response to microbiota, honeybees produce antimicrobial peptides (AMPs) as a major part of their non-specific defense system against infections. Honeybee-produced AMPs include abaecin, apidaecin, defensin, and hymenoptaecin (Keitel et al., 2013; Horak et al., 2020). To advance the development of the honeybee PD model, future studies are necessary to explore the role played by AMPs in the microbiota-gut-brain axis.

The elevated alpha diversity in the CV group suggests a richer microbial community within the gut compared to the HC and PD groups, reflecting a healthier status in the gut microbial ecosystem of the CV group. However, it is also possible that this increased alpha diversity reflects that the microbiota from bees may colonize bees more effectively than that from humans. The honeybee microbiota plays a pivotal role in preserving the individual’s health, and disturbance to the microbiota renders the insect susceptible to a range of problems (Nowak et al., 2021). Various studies have substantiated the presence of microbiota-gut-brain axis, signifying that gut microorganisms instigate modifications in neurophysiology and induce alterations in the behavior of insect hosts by biogenic amines such as serotonin, octopamine, and dopamine (Westfall et al., 2018; Leger and McFrederick, 2020).

Previous studies using gnotobiotic bees have revealed that gut microbiota alters both brain and behavioral phenotypes. Microbiota-free bees have been reported to show deficits in appetitive olfactory learning and memory, sucrose responsiveness, and social interactions when compared to CV bees (Cabirol et al., 2023). Our results showed that the differentially abundant microbiota between groups may impair the hosts’ health in several ways. *M. morganii* and *E. ramosum*, the relative abundance of which increased in the HC group, are recognized as gut commensal microbiota in humans and other mammals (Zrelovs et al., 2022) and may not cause impairment to the honeybees. As for gut microbiota enriched in the PD group, *D. longicatena* can produce formate, a toxic metabolite inhibiting mitochondrial cytochrome C oxidase (Nicholls, 1976). Moreover, formate was found to increase in the serum of PD patients (Nagesh Babu et al., 2018). *C. aerofaciens* induces the expression of IL-17 and the chemokines CXCL1 and CXCL5, causing a loss of gut epithelial integrity under pro-inflammatory conditions (Kalinkovich and Livshits, 2019). *B. obeum*, and *D. longicatena* were also enriched in dystonia subjects (Ma et al., 2021), a disease with neuronal damage and neurotransmitter abnormalities. In summary, these findings indicate dysbiosis of the gut microbiota in the PD group, and it may negatively impact host health through various pathways, including modulation of the host immune system, generation of toxic metabolites, and damage to gut mucosal integrity.

For the predicted functional pathways, lysine biosynthesis was potentially downregulated in the PD group. Lysine, an α-amino acid that is a precursor to many proteins, is essential in humans and cannot be synthesized by humans. The pathways involving methane metabolism and H_2_S production were elevated in the PD group. Emerging evidence suggests that methane delays intestinal transit, possibly acting as a neuromuscular transmitter. Additionally, methane has been epidemiologically and clinically associated with constipation-related diseases, such as constipation-predominant irritable bowel syndrome and chronic constipation (Pimentel et al., 2006; Triantafyllou et al., 2014). Previous studies have shown that methane slows down bowel movements, which indicates that the abnormal methane metabolism observed in our results may be related to the common symptom of constipation in patients with PD (Jang et al., 2020). H_2_S is produced by microbes of the colon (Paul and Snyder, 2015). H_2_S has cytoprotective properties by maintaining gut mucus integrity but is toxic to the host at high concentrations (Buret et al., 2022). High H_2_S concentrations are toxic to cells, causing the inhibition of the cytochrome oxidase, a hemeprotein that is the last enzyme of the electron transport chain in the mitochondria. In addition, H_2_S induces the release of cytochrome C protein from the mitochondrial membrane, an event thought to be associated with the etiopathogenesis of PD (Murros, 2022).

The study has the following limitations: first, further metabolomics and proteomics analyses are needed to identify neuroactive metabolites and proteins in the host gut, hemolymph, and brain, and to reveal the link between such substances and the gut microbiota in a strain-specific manner. Second, patients recruited in the study are on medications, which may be a confounder influencing the results. Third, there are inherent differences in the anatomical and physiological structures between honeybees and humans, which necessitate caution when interpreting findings from the bee model and applying them to human clinical contexts. Fourth, some bacterial taxa from human donors may have a higher propensity for colonizing the bee gut, potentially leading to a shift in microbial composition. Fifth, this is the first attempt to use honeybees as PD model animals, and more repeated experiments are needed to verify the practicability and credibility of the model.

In conclusion, despite the above limitations, we found that it was feasible to use rotenone and gut microbiota to construct a PD honeybee model and that the pathogenesis of the microbiota-gut-brain axis might also exist in the PD honeybee model.

## Data availability statement

The raw sequence data reported in this paper have been deposited in the Genome Sequence Archive https://ngdc.cncb.ac.cn/gsa (Genomics, Proteomics & Bioinformatics 2021). The data can be accessed in the National Genomics Data Center (Nucleic Acids Res. 2022), China National Center for Bioinformation/Beijing Institute of Genomics, Chinese Academy of Sciences, under accession number CRA014586.

## Ethics statement

The studies involving humans were approved by the Ethics Committee of Chinese PLA General Hospital (No. S2019-061-02). The studies were conducted in accordance with the local legislation and institutional requirements. The participants provided their written informed consent to participate in this study. The manuscript presents research on animals that do not require ethical approval for their study.

## Author contributions

JZ: Formal analysis, Investigation, Methodology, Visualization, Writing – original draft. YL: Methodology, Writing – review & editing. JY: Methodology, Writing – review & editing. RC: Methodology, Writing – review & editing. MX: Methodology, Writing – review & editing. GZ: Methodology, Writing – review & editing. JM: Methodology, Writing – review & editing. DL: Methodology, Writing – review & editing. ZM: Conceptualization, Supervision, Writing – review & editing. YY: Conceptualization, Funding acquisition, Supervision, Writing – review & editing.

## Footnotes

^1^ https://cloud.majorbio.com/page/tools/

## Supplementary material

SUPPLEMENTARY FIGURE S1 Dopamine concentrations in the brains of honeybees. HC: *n* = 9, CV: *n* = 3, PD: *n* = 15, ROT: *n* = 3. ns, not significant. Statistical analysis was conducted using the Mann–Whitney *U* test.

SUPPLEMENTARY FIGURE S2 Principal coordinate analysis (PCoA) plots based on the Bray–Curtis distance. **(A)** PCoA on species level. **(B)** PCoA on genus level.

SUPPLEMENTARY FIGURE S3 The alpha diversity analysis of gut microbiota of honeybees. **(A)** Kruskal–Wallis *H* test for Chao index. **(B)** Kruskal–Wallis *H* test for Shannon index.

SUPPLEMENTARY FIGURE S4 The beta diversity analysis of gut microbiota of honeybees. **(A)** Principal component analysis (PCA) on species level. **(B)** PCA on genus level.
