# Supplementary material for: Gut microbiota from patients with Parkinson’s disease causes motor deficits in honeybees
Source: Front Microbiol. 2024 Jul 12;15:1418857. doi: 10.3389/fmicb.2024.1418857 (PMC11272988; doi:10.3389/fmicb.2024.1418857)
Supplement: Supplementary file 1 [file Table_1.DOCX]

| Table S1. Demographic and clinical data of human donors | | | | | | |
| --- | --- | --- | --- | --- | --- | --- |
| No. | Age | Sex | Disease  duration | Hoehn-Yahr stage | UPDRS part III | Constipation scoring system |
| PD1 | 76 | Female | 6 | 4.0 | 45 | 11 |
| PD2 | 71 | Male | 6 | 4.0 | 80 | 16 |
| PD3 | 53 | Female | 8 | 1.0 | 29 | 4 |
| PD4 | 71 | Female | 6 | 4.0 | 53 | 11 |
| PD5 | 63 | Female | 4 | 3.0 | 37 | 16 |
| HC1 | 63 | Male | NA | NA | NA | NA |
| HC2 | 50 | Female | NA | NA | NA | NA |
| HC3 | 67 | Female | NA | NA | NA | NA |
| UPDRS: unified Parkinson’s disease rating scale; PD: Parkinson’s disease; HC: heathy control. | | | | | | |
