# Supplementary figures and images for: Gut microbiota from patients with Parkinson’s disease causes motor deficits in honeybees
Source: Front Microbiol. 2024 Jul 12;15:1418857. doi: 10.3389/fmicb.2024.1418857 (PMC11272988; doi:10.3389/fmicb.2024.1418857)

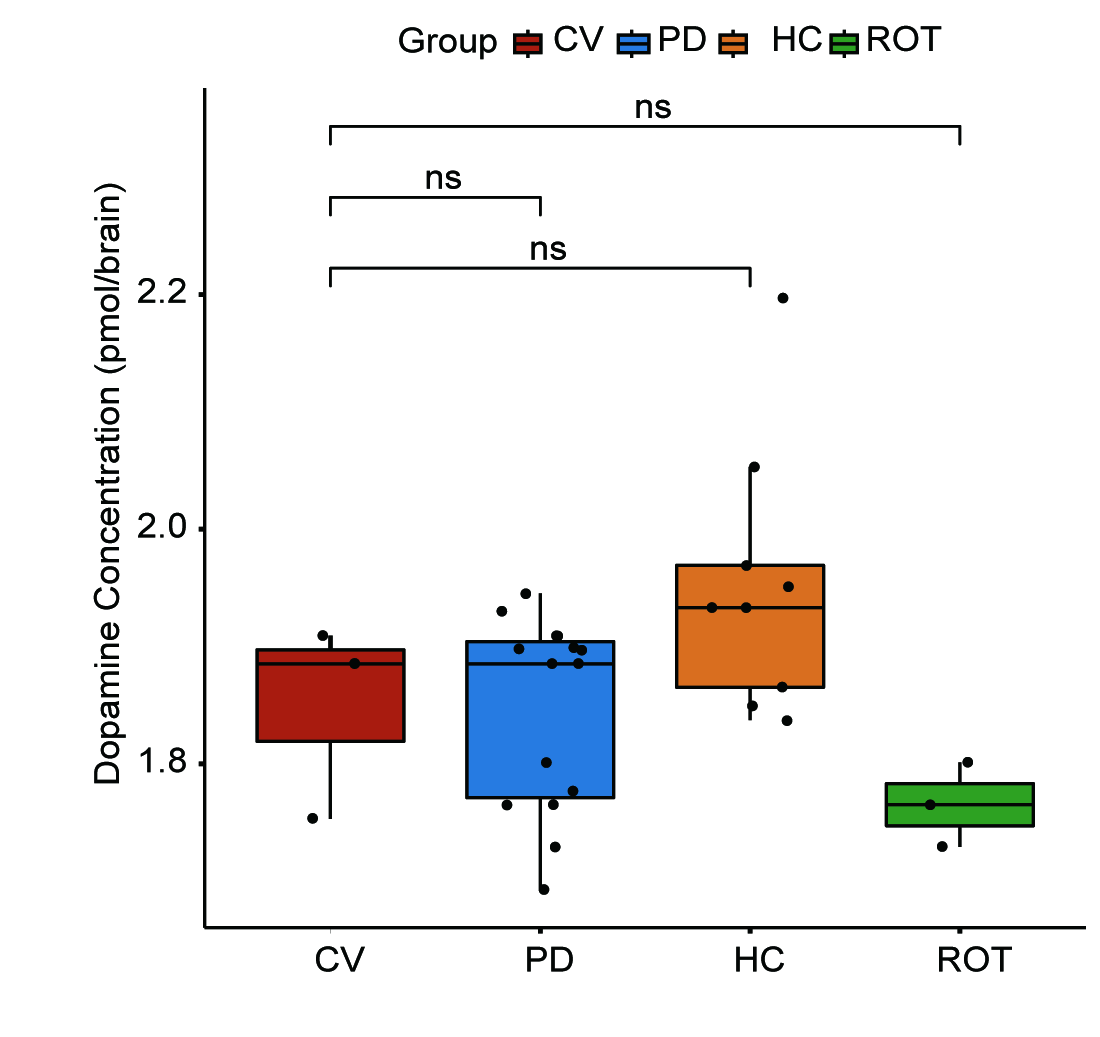

Supplement: SUPPLEMENTARY FIGURE S1 — Dopamine concentrations in the brains of honeybees. HC: n = 9, CV: n = 3, PD: n = 15, ROT: n = 3. ns, not significant. Statistical analysis was conducted using the Mann–Whitney U test. [file Image_1.TIF]

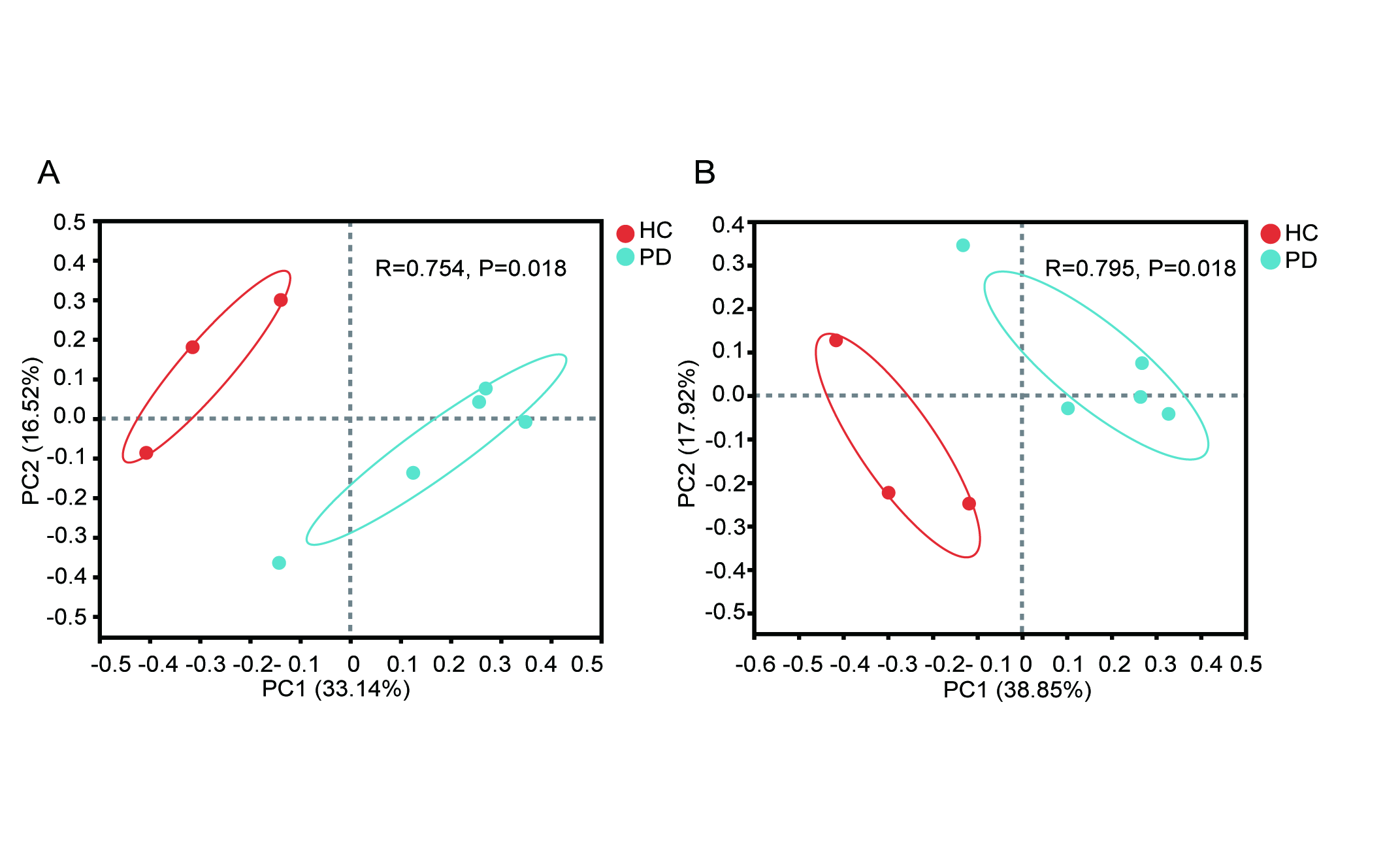

Supplement: SUPPLEMENTARY FIGURE S2 — Principal coordinate analysis (PCoA) plots based on the Bray–Curtis distance. (A) PCoA on species level. (B) PCoA on genus level. [file Image_2.TIF]

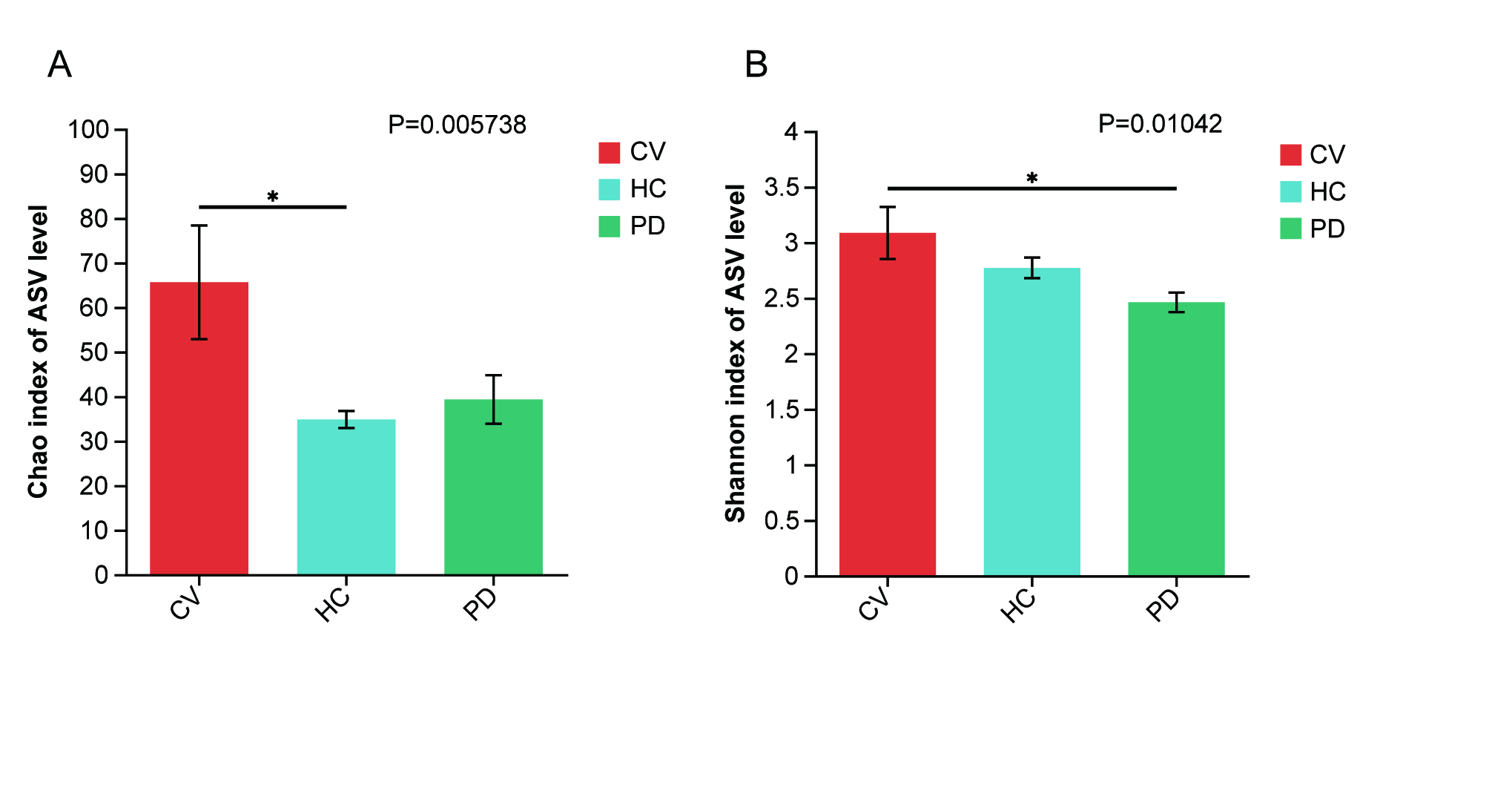

Supplement: SUPPLEMENTARY FIGURE S3 — The alpha diversity analysis of gut microbiota of honeybees. (A) Kruskal–Wallis H test for Chao index. (B) Kruskal–Wallis H test for Shannon index. [file Image_3.TIF]

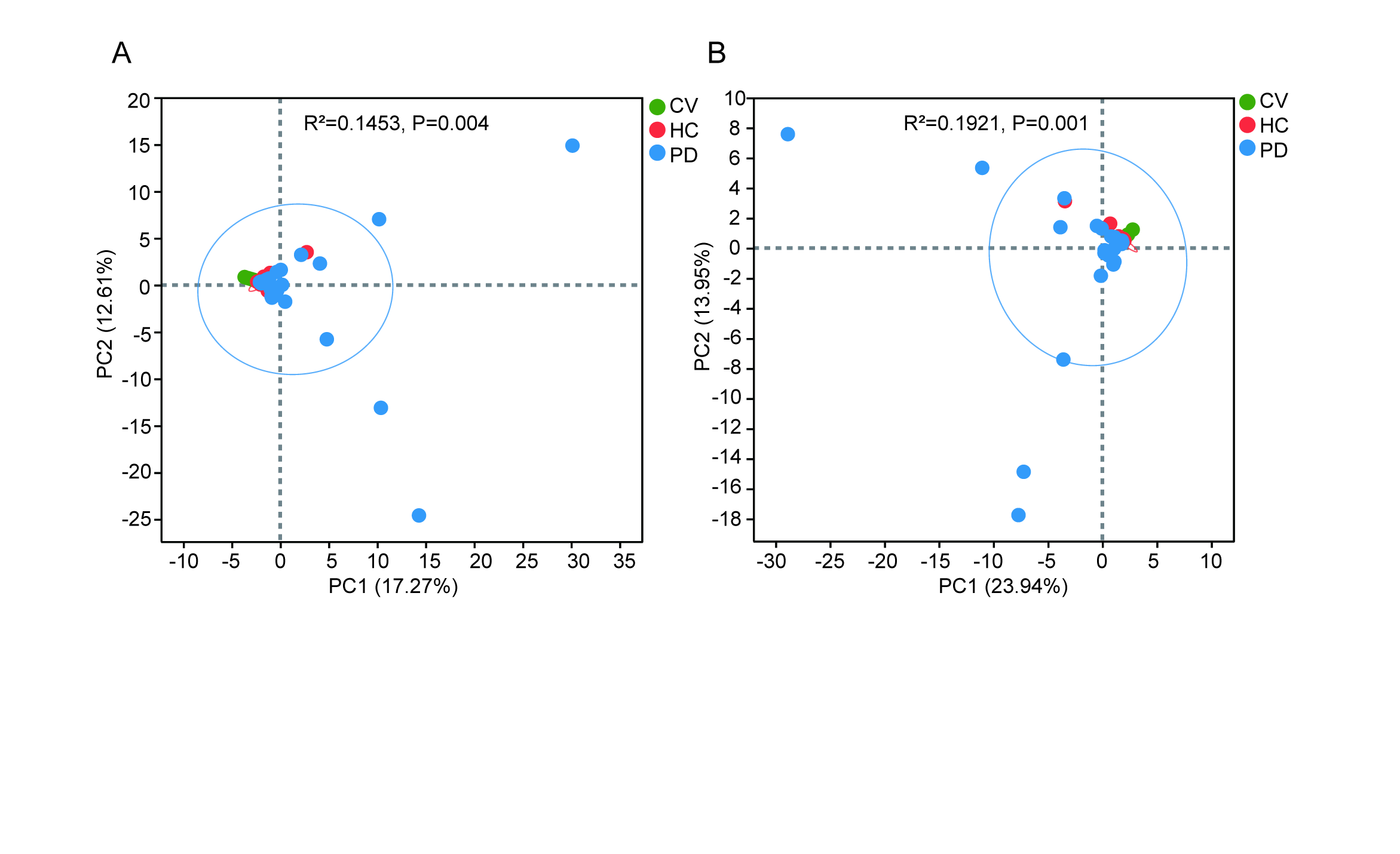

Supplement: SUPPLEMENTARY FIGURE S4 — The beta diversity analysis of gut microbiota of honeybees. (A) Principal component analysis (PCA) on species level. (B) PCA on genus level. [file Image_4.TIF]
